# Cerebrospinal Fluid Genetics Enhance Risk Stratification in Bipolar Disorder

**DOI:** 10.1002/mco2.70629

**Published:** 2026-02-26

**Authors:** Yu Feng, Xiaonan Guo, Peng Huang, Xiaolong Ji, Ningning Jia, Sheng Yang, Shaohua Hu

**Affiliations:** ^1^ Department of Psychiatry the First Affiliated Hospital Zhejiang University School of Medicine Zhejiang China; ^2^ Department of Epidemiology Centre for Global Health School of Public Health National Vaccine Innovation Platform Key Laboratory of Public Health Safety and Emergency Prevention and Control Technology of Higher Education Institutions in Jiangsu Province Nanjing Medical University Nanjing China; ^3^ Liangzhu Laboratory Hangzhou China; ^4^ Division of Pediatric Pulmonary Medicine, UPMC Children's Hospital of Pittsburgh University of Pittsburgh Pittsburgh Pennsylvania USA; ^5^ Nanhu Brain‐Computer Interface Institute Hangzhou China; ^6^ Zhejiang University School of Medicine Hangzhou China; ^7^ The Zhejiang Key Laboratory of Precision Psychiatry Hangzhou China; ^8^ The State Key Lab of Brain‐Machine Intelligence Zhejiang University Hangzhou China; ^9^ MOE Frontier Science Center for Brain Science and Brain‐Machine Integration Zhejiang University School of Medicine Hangzhou China; ^10^ Brain Research Institute of Zhejiang University Hangzhou China; ^11^ Zhejiang Engineering Center For Mathematical Mental Health Hangzhou China

**Keywords:** bipolar disorder, cerebrospinal fluid, GWAS, risk stratification

## Abstract

Bipolar disorder (BD) research confronts challenges: blood‐based biomarkers offer limited insights into neurobiology, while cerebrospinal fluid (CSF) collection is clinically unusual. Linking genetic susceptibility to pathophysiology remains crucial for biologically informed risk stratification. We integrated cohort data and genome‐wide association study (GWAS) summary statistics: the largest BD meta‐analysis, CSF multi‐omics profiles including 3107 proteomic and 2602 metabolomic participants, and a validation cohort of 247,834 UK Biobank participants. Unsupervised clustering revealed four single‐nucleotide variant (SNV) clusters: metabolic‐imbalance, metabolic‐active, human leukocyte antigen (HLA)+immune, and HLA‐immune. These clusters exhibited distinct clinical features, with the metabolic‐imbalance cluster showing multi‐directional associations with 21 psychiatric traits, while the HLA‐immune cluster was associated with emotional instability in BD patients (odds ratio [OR] = 1.14, *p* = 0.027). The optimized multimodal cluster‐specific polygenic risk scores (PRS) model significantly outperformed clinical‐only prediction factors (C‐index = 0.77), with the metabolic‐imbalance PRS contributing a 22.6% incremental predictive value (hazard ratio [HR] = 1.23, 95% CI: 1.04–1.45, *p* = 0.016). Risk reclassification showed an 84% reduction in false‐negative rates in the low‐risk subgroup, identifying a high‐risk layer with a 17.6‐fold increased BD incidence. Altogether, genetically informed substitutes for CSF biomarkers emerged as a scalable tool for risk prediction, overcoming the barriers of CSF collection while capturing neurobiological heterogeneity.

## Graphical abstract

This study introduced a novel genetic proxy strategy that connects the CSF biomarkers with the clinical applications in BD research. First, it delineated a genetically anchored CSF biomarker network. Second, it demonstrated the clinical utility of multimodal PRS. Finally, it provided a scalable framework for risk stratification. These insights pave the way for precision monitoring and therapeutic strategies tailored to the heterogeneous pathophysiology of BD.

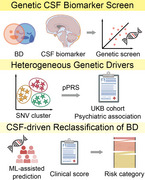

## 1 Introduction

Bipolar disorder (BD) presents a significant challenge in the field of global mental health, with a lifetime prevalence of 1%–2% and considerable clinical heterogeneity [1, 2]. Although twin studies estimate the heritability of BD to be between 60% and 85%, variations identified through genome‐wide association studies (GWAS) account for less than 20% of the disease risk [3, 4]. This underscores a significant gap in understanding how genetic susceptibility translates into neurobiological mechanisms.

Existing biomarker studies predominantly focus on peripheral blood, revealing associations with inflammatory cytokines and metabolic markers [5, 6, 7, 8]. However, due to the blood–brain barrier, blood biomarkers have inherent limitations in reflecting central nervous system (CNS) pathophysiology, potentially decoupling from key disease‐related signals.

Recent advancements in cerebrospinal fluid (CSF) proteomics and metabolomics have provided unprecedented opportunities to directly investigate CNS pathways [9, 10]. Compared to peripheral indicators, CSF biomarkers offer higher specificity for neuropsychiatric disorders. Emerging evidence suggests that dysregulation of synaptic proteins, neuroinflammatory mediators, and energy metabolism intermediates is implicated in the pathogenesis of BD [11, 12, 13]. However, the clinical inaccessibility of CSF samples remains a significant barrier in BD research [14], limiting biomarker discovery to small‐scale cross‐sectional studies and severely constraining translational applications.

We hypothesized that genetic variants predisposing individuals to BD exert coordinated effects on CSF biomarkers that reflect immune–metabolic dysregulation. To test this hypothesis, we developed a novel integrative framework that bridged molecular and population genetics through functional analysis. Using Mendelian randomization (MR), we combined the largest BD GWAS and CSF multi‐omics datasets to identify CSF biomarkers genetically associated with BD. We then applied proteome‐wide and metabolome‐wide association studies (PWAS/MWAS) followed by unsupervised k‐means clustering of BD‐associated variants to delineate biologically coherent modules representing shared molecular axes. For each cluster, we constructed polygenic risk scores (PRS) to translate these CSF‐derived molecular axes into individual‐level genetic risk estimates. Finally, by integrating clinical data from the UK Biobank (UKB), we demonstrated that the synergistic effect of CSF biomarker–inferred PRSs and electronic health record information enables biology‐based risk stratification for BD, providing a mechanistically informed and non‐invasive framework for precision psychiatry.

## 2 Results

### 2.1 BD Biomarker Screening in CSF Proteins and Metabolites

Using PWAS/MWAS‐FUSION, we identified 20 metabolites, 75% of which are amino acids, and 61 CSF proteins associated with BD (Tables S1 and S2). Four metabolites and 27 proteins were validated through MR analysis (Tables S3 and S4). For colocalization analysis, we first identified 1418 index pQTL associations for the 61 proteins and 147 index mQTL associations for the 20 metabolites (Tables S5 and S6). Bayesian colocalization revealed that 32 proteins colocalize with BD risk loci (Table S7), while among metabolites, 1‐palmitoyl‐2‐dihomo‐linolenoyl‐GPC showed colocalization with BD (Table S8). This metabolite also exhibited a significant positive association with BD risk in the MR analysis.

Enrichment analysis revealed these biomarkers are linked to key immune pathways, including antigen processing and presentation (particularly MHC class I and Ib pathways), T‐cell‐mediated immune response, and various metabolic processes regulating immune responses (Table S9). These findings suggested immune regulation and metabolic pathways in the pathogenesis of BD at the CSF level.

### 2.2 Heterogeneous Genetic Drivers of BD Biomarkers

**FIGURE 1:**
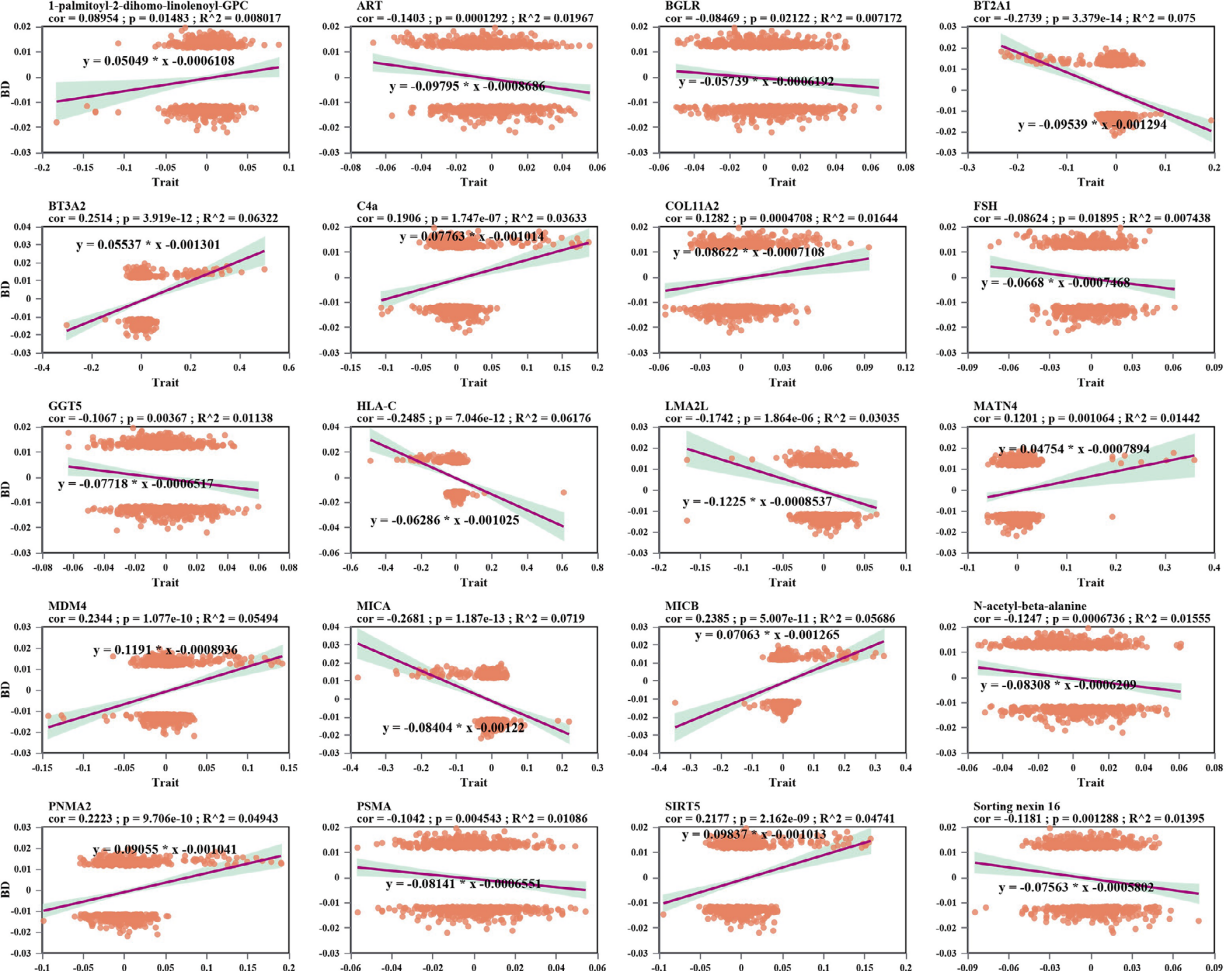
Correlation between BD risk variants and CSF biomarkers. Alignment of 31 CSF biomarkers with 740 BD risk alleles from SNVs. The z‐scores were adjusted based on sample size, and Pearson correlation analyses were performed between each of the 31 CSF biomarkers and BD SNVs. This figure presented only the results with a *p*‐value < 0.05.

To unravel the contribution of genetic factors to BD biomarkers, we classified 740 BD‐associated SNVs and examined their correlation with biomarkers (Table S10). Pearson correlation analyses were performed between 31 biomarkers and BD‐associated SNVs, leading to the inclusion of 20 biomarkers (two metabolites and 18 proteins) for clustering analysis (Figure 1 and Figure S1). Based on the clustering results, four distinct BD SNV clusters were identified (Figure S2). A linear regression model was used to test the association between each biomarker and the BD SNVs within the clusters, revealing that these SNVs had coordinated effects on CSF biomarkers (Table S11).

**FIGURE 2:**
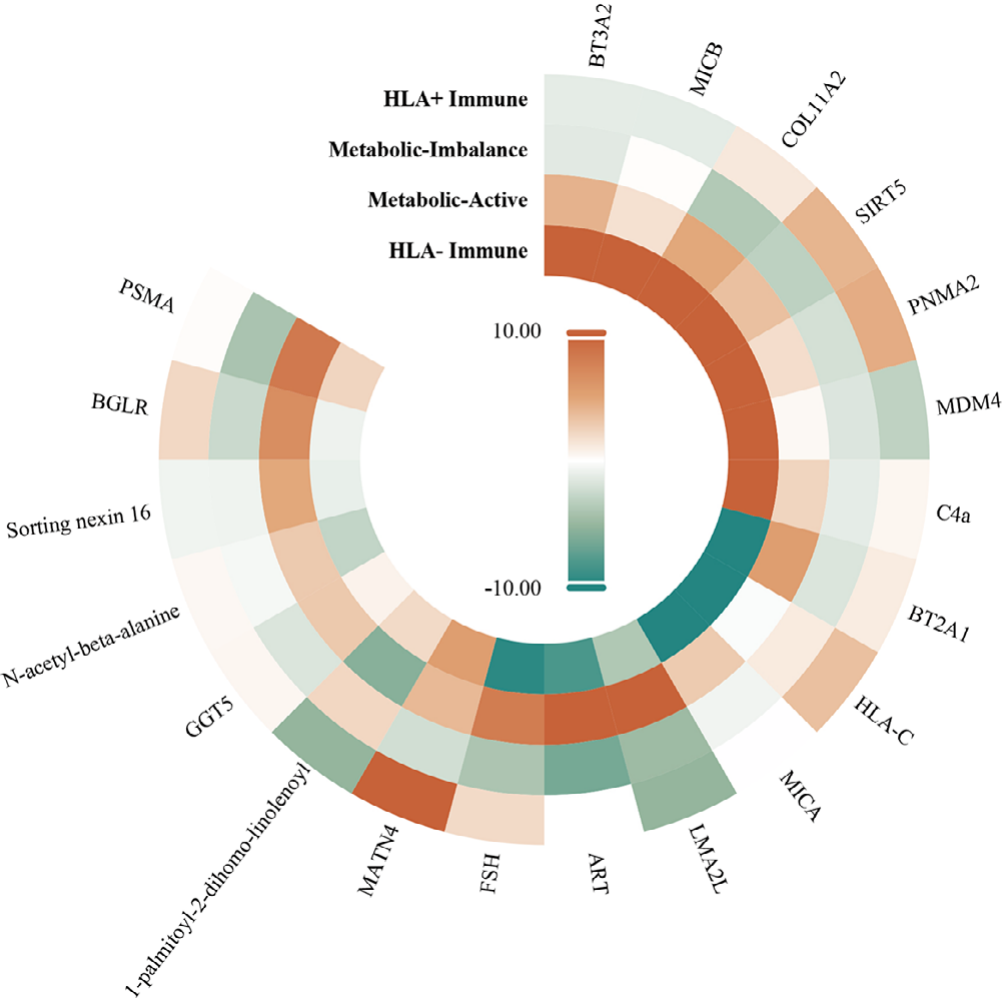
Cluster‐specific genetic effects on CSF biomarkers. Associations between each biomarker and BD SNVs within each cluster were tested using a linear regression model. Each column represented a different cluster, and each row corresponds to a CSF biomarker. Each cell represented the *Z*‐score (Estimate/St. Error) for the association between the biomarker and the BD SNVs assigned to that cluster.

The metabolic‐imbalance cluster (C1) exhibited downregulation of mitochondrial regulators (*SIRT5*: *Z* = −3.12, *p* = 1.85 × 10^−^^3^) and gonadotropin signaling (*FSH*: *Z* = −3.96, *p* = 8.36 × 10^−^^5^) (Figure 2) [15, 16], which reflected dysregulation of the hypothalamic‐pituitary axis in BD [17, 18, 19]. The metabolic‐active cluster (C2) showed contrasting effects, combining phosphatidylcholine (1‐palmitoyl‐2‐dihomo‐linolenoyl‐GPC: *Z* = −5.64, *p* = 2.46 × 10^−^^8^) with neuropeptide hormone activity (*ART*: *Z* = 12.53, *p* = 8.65 × 10^−^^3^ ^3^) and acetyl‐aspartate‐glutamate binding (*PSMA*: *Z* = 8.20, *p* = 1.07 × 10^−^^1^ ^5^), suggesting compensatory inflammatory signaling [20, 21]. The HLA+immune cluster (C3) exhibited moderate MHC‐I activation (*HLA‐C*: *Z* = 3.41, *p* = 6.73 × 10^−^^4^) and extracellular matrix remodeling (*MATN4*: *Z* = 14.94, *p* = 2.94 × 10^−^^4^ ^4^), which indicated subclinical neuroinflammation [22, 23].

Notably, the HLA‐immune cluster (C4) displayed extreme polarization of immune checkpoints, characterized by both *HLA‐C* suppression (*Z* = −25.53, *p* = 1.74 × 10^−^^1^ ^0^ ^3^) and activation of natural killer ligands (*MICB*: Z = 31.01, *p* = 1.04 × 10^−^^1^ ^3^ ^5^) [24], along with contradictory metabolic reprogramming (*SIRT5*: *Z* = 25.93, *p* = 8.60 × 10^−^^1^ ^0^ ^6^). The extraordinary magnitude of effect (*Z*: −35.87 to +39.27) and lipid–protein interactions suggested that the genetic architecture of BD primarily involved immune‐invading cell states, accompanied by mitochondrial stress responses, emphasizing observed metabolic‐immune duality in BD pathophysiology.

### 2.3 Association Between Cluster‐Specific PRS and Psychiatric Disorders

**FIGURE 3:**
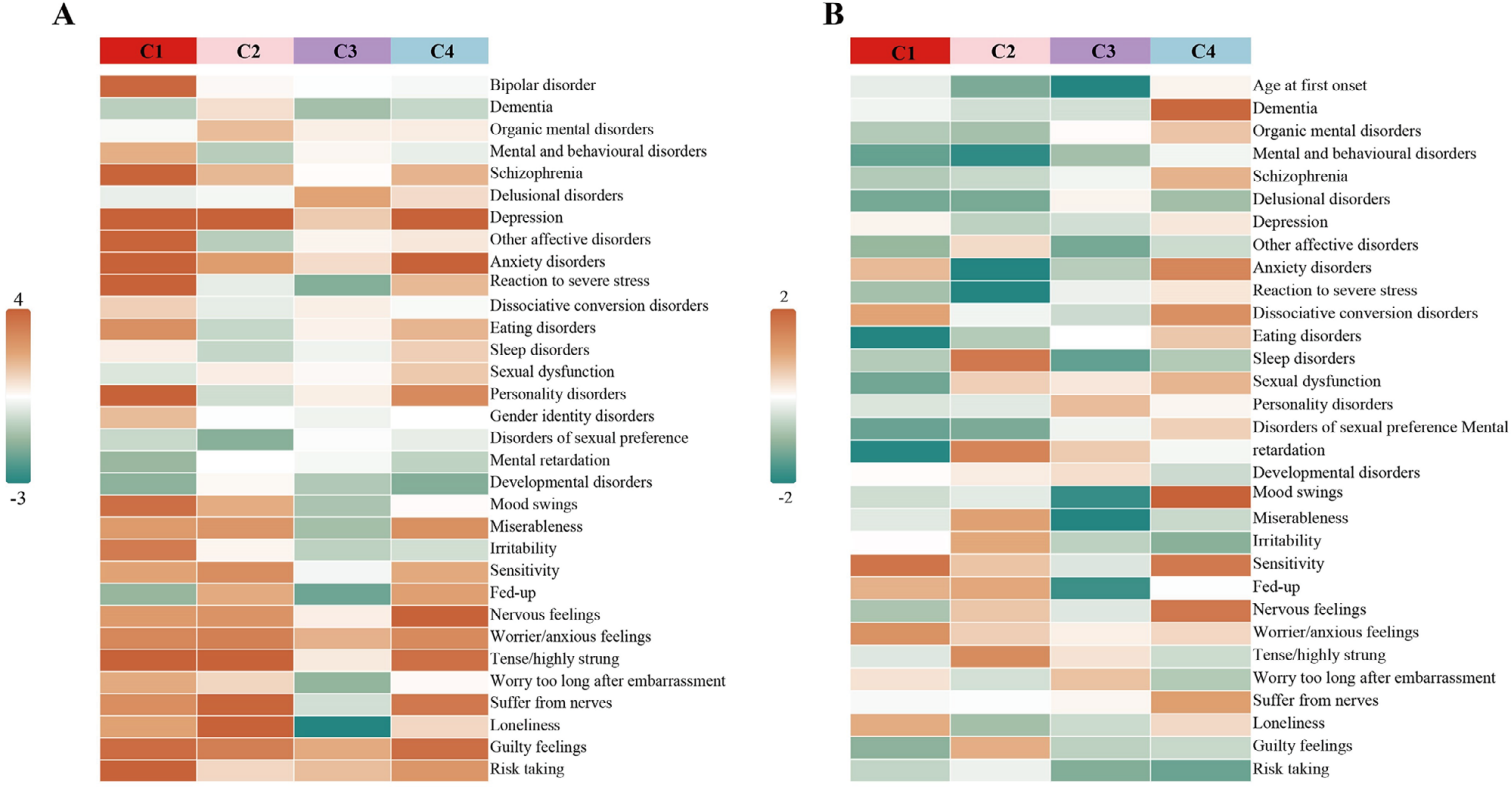
Associations between cluster‐specific PRS and psychiatric phenotypes. After adjusting for the first five PCs, the BD effect size of each cluster SNV was multiplied by the genotypes of UKB individuals to calculate the cluster‐specific PRS, which was then standardized to have a mean of zero and unit variance. (A) The association between the four cluster‐specific PRSs and psychiatric‐related phenotypes was tested. (B) The association between the four cluster‐specific PRSs and BD‐related comorbid psychiatric symptoms was tested.

Given the high and heterogeneous comorbidity of psychiatric disorders in BD [25], we examined the association between cluster‐specific PRS and psychiatric conditions as well as mental health traits in general populations (mean age, 56.7 years; 133,053 [54%] female). Generalized linear models revealed distinct cross‐diagnostic associations across the four clusters, with stratified effect sizes reflecting the perturbation patterns of their biomarkers (Figure 3A and Table S12). The metabolic‐imbalance cluster showed the broadest pleiotropy (21 associated phenotypes), elevating risks for BD (OR = 1.18, FDR = 3.95 × 10^−^^4^), schizophrenia (OR = 1.18, FDR = 3.03 × 10^−^^4^), and depression (OR = 1.05, FDR = 1.80 × 10^−^^12^), while predicting dimensional phenotypes, including risky behaviors (OR = 1.037, FDR = 8.05 × 10^−^^13^) and mood swings (OR = 1.015, FDR = 7.53 × 10^−^^4^) (Figures S3 and S4). The metabolic‐active cluster exhibited selective associations with anxiety‐related traits, especially high tension (OR = 1.025, FDR = 1.09 × 10^−^^4^) and social withdrawal (loneliness OR = 1.022, FDR = 4.49 × 10^−^^4^). The HLA+immune cluster showed contradictory effects, increasing the risk of delusional disorders (OR = 1.14, *p* = 2.26 × 10^−^^2^) while decreasing susceptibility to loneliness (OR = 0.983, FDR = 4.13 × 10^−^^2^). However, it was noteworthy that the association with delusional disorders did not remain significant after Benjamini–Hochberg correction (FDR  =  0.37). The HLA‐immune cluster had a concentrated effect on mood and personality, showing positive associations with personality disorders (OR = 1.16, FDR = 1.40 × 10^−^^2^) and generalized anxiety (OR = 1.035, *p* = 8.28 × 10^−^^5^).

**FIGURE 4:**
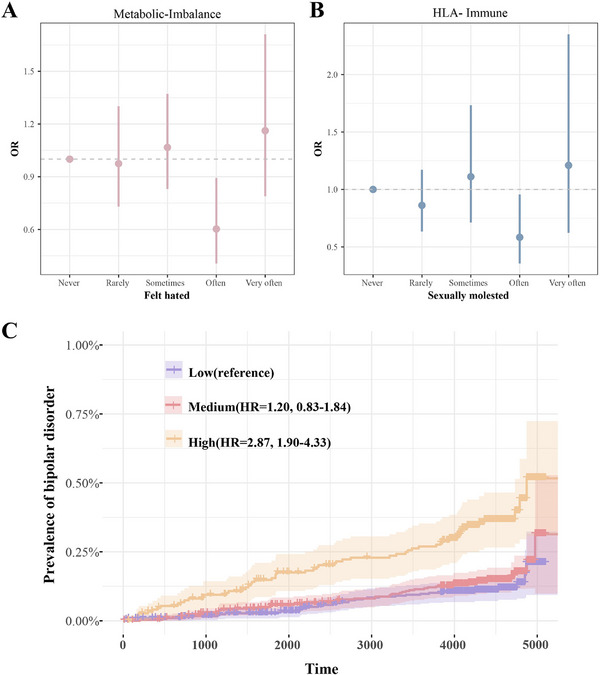
Interaction effects and genetic risk stratification based on multimodal PRS models. (A) The interaction between Felt hated and C1 PRS on the association with BD using “Never true” as the reference group. (B) The interaction between sexually molested and C4 PRS on the association with BD using “Never true” as the reference group. (C) Based on the distribution of the multimodal PRS model, participants were divided into low, medium, and high genetic risk groups. Cox regression was conducted, with the low genetic risk group used as the reference group.

Childhood trauma and cluster‐specific PRS interaction analyses revealed context‐dependent effects on BD risk (Table S13). For the “felt hated” item, individuals carrying the metabolic imbalance cluster showed a significantly reduced risk of BD in the “often” group (OR = 0.60, *p* = 0.012), while no significant associations were observed in other groups (Figure 4A). Similarly, for the “sexually molested” item, HLA‐immune cluster carriers in the “often” group demonstrated a significantly lower BD risk (OR = 0.58, *p* = 0.033) (Figure 4B). These findings suggest a potential protective effect of genetic risk within specific psychosocial stress contexts. However, it is important to note that these apparent protective associations may not necessarily reflect true biological resilience but could instead result from selection bias, collider effects, or differential reporting of trauma exposure.

### 2.4 Association Between Cluster‐Specific PRS and BD Comorbidities

Cluster‐specific PRS revealed distinct patterns of BD comorbidities in a cohort of 1593 BD patients (mean age, 47.1 years; 922 [58%] female) (Figure 3B and Table S14). The metabolic‐imbalance cluster showed protection against eating disorders in BD patients (OR = 0.29, *p* = 3.58 × 10^−^^5^), coordinated with downregulation of *SIRT5* and *ART* (Figure S5) [26, 27]. The metabolic‐active cluster demonstrated moderate protective associations with anxiety (OR = 0.88, *p* = 1.48 × 10^−^^2^) and stress responses (OR = 0.81, *p* = 1.74 × 10^−^^2^) in BD patients. The HLA+immune cluster accelerated BD onset (OR = 0.38, *p* = 1.65 × 10^−^^2^) and alleviated psychological distress (miserableness OR = 0.88, *p* = 1.95 × 10^−^^2^), consistent with its moderate immune activation potentially accelerating neurodevelopmental trajectories while buffering emotional dysregulation. The HLA‐immune cluster promoted emotional instability in BD patients (OR = 1.14, *p* = 2.69 × 10^−^^2^), which aligns with its extreme immune checkpoint dysregulation and mitochondrial stress response observed in biomarker analyses.

### 2.5 Multimodal PRS Cox Model

Cluster‐specific PRS demonstrated varied predictive effects for BD. The metabolic‐imbalance cluster (C index = 0.58) provided incremental value over the traditional overall PRS (C index = 0.56) and was associated with a 22.6% increase in BD risk (HR = 1.23, 1.04–1.45, *p* = 1.56 × 10^−2^), outperforming other clusters (Figures S6–S8 and Tables S15 and S16).

To integrate the genetic prediction of CSF biomarkers and the identified cluster‐specific PRS (i.e., metabolic‐imbalance cluster) for BD risk prediction, we established the multimodal prediction framework through ML. Ridge regression‐optimized multimodal model, integrating 12 biomarker PRSs and the metabolic‐imbalance cluster PRS, improved prediction by 11% over the baseline model (C index = 0.65), amplifying BD risk (HR = 1.72, 1.50–1.97, *p* = 4.92 × 10^−15^), approaching plasma proteomic performance (C‐index = 0.69) (Figure S9) [28]. Participants stratified by multimodal PRS quintiles demonstrated that individuals with high genetic risk had a 187% higher relative risk of BD compared to those with low genetic risk (HR = 2.87, 1.90–4.33, *p* = 5.93 × 10^−^^7^) (Figure 4C). Considering the integration of clinical scores, although clinical indicators showed strong independent predictive ability (C‐index = 0.72), the clinical‐ML combined model (Clinical Score + Ridge) performed the best (C‐index = 0.77; HR = 1.80, 1.67–1.94, *p* = 1.38 × 10^−51^), with a 169.4 reduction in AIC compared to the baseline. In addition, we found that the Clinical Score + traditional overall PRS model (C‐index = 0.718) performed slightly worse than the Clinical Score + metabolic‐imbalance cluster PRS model (C‐index = 0.723) and was inferior to the clinical–ML combined model. These results suggested that the multimodal integration of clinical scores, CSF biomarkers, and cluster‐specific genetics offered superior risk prediction.

We further applied the above models to distinguish between different BD subtypes, including 293 BD‐I and 376 BD‐II patients. Unfortunately, the other models did not show significant improvement compared to the BD score model (Table S17). Among the PRS‐based models, only the metabolic‐imbalance cluster PRS model demonstrated a modest improvement of approximately 5% over the baseline model. This limited gain is likely attributable to the relatively small sample size of each BD subtype and their high genetic correlation, which reduces the discriminative power of subtype‐specific risk prediction.

### 2.6 Reclassification of Risk Using “Clinical Score × Hybrid Risk Factor”

**FIGURE 5:**
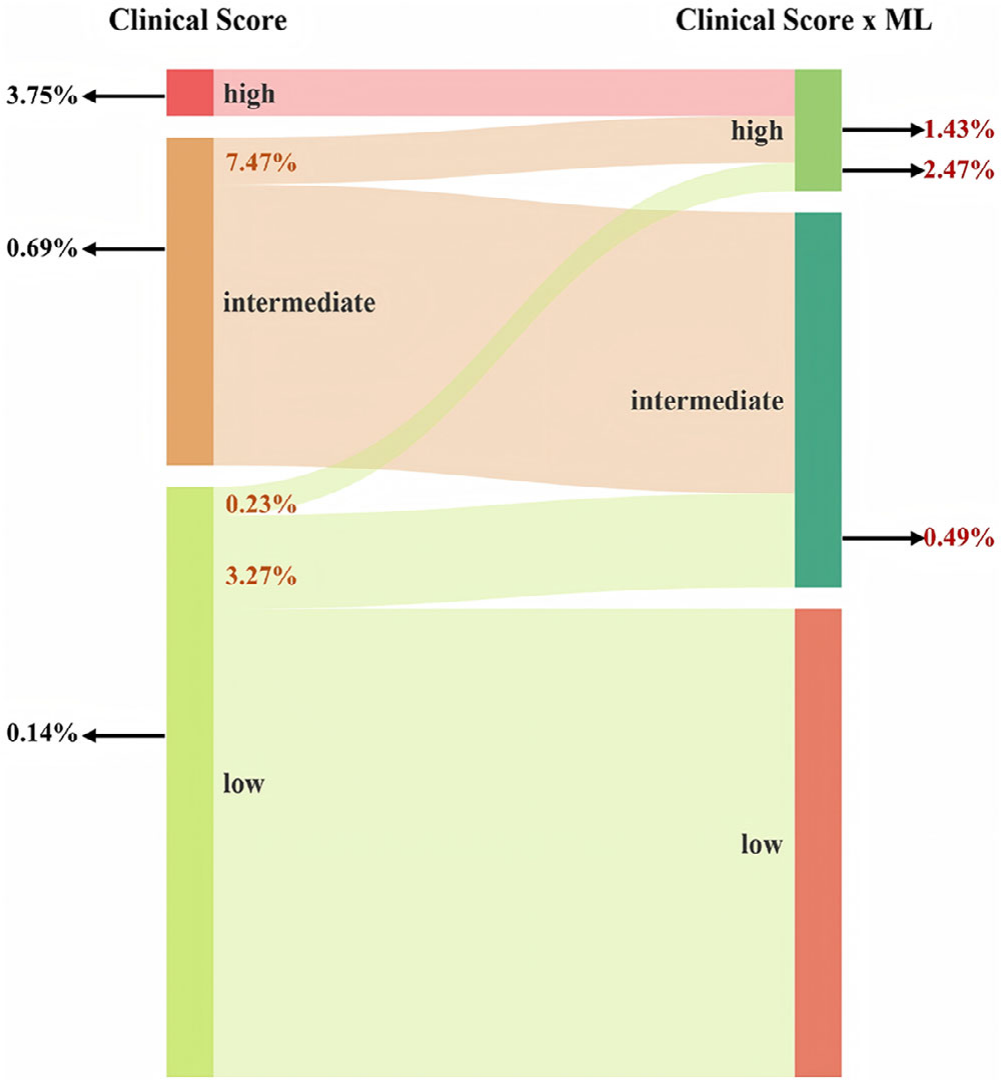
Risk reclassification of bipolar disorder using a multiplicative clinical–genetic model. Comparison of the multiplicative model with traditional clinical scales for BD risk categorization.

We developed a clinical risk classification model, where individuals were reclassified by multiplying the absolute clinical risk derived from the clinical score with the corresponding ML‐derived hybrid risk factor (Clinical Score × Hybrid Risk Factor). Compared to using the clinical score alone, the multiplicative model provided higher precision in identifying gradations of BD susceptibility (Figure 5 and Figure S10).

In the low‐risk clinical category, the multiplicative model reclassified 3.5% of individuals into higher risk levels, including an intermediate‐risk group with a 3.5‐fold increased incidence and a high‐risk group with a 17.6‐fold increased risk (2.47%), yielding results similar to global BD prevalence (Table S18). For the intermediate‐risk clinical group, the model enhanced stratification through 7.47% high‐risk reclassification with a doubling incidence rate, while 92.53% of moderate‐risk individuals maintained accurate probability estimates. Note that the model‐maintained calibration in the high‐risk group, without misclassification dilution for previously classified individuals.

The combined multiplicative risk demonstrated strong gradient discrimination, with ML‐adjusted high‐risk groups showing 20.6‐fold (2.47%/0.12%) and 5.4‐fold (1.43%/0.62%) increases in risk relative to the baseline clinical strata. This biological‐genetic‐clinical synergy highlighted that traditional phenotype analysis captured population‐level risk variation, while ML‐derived genetic biomarker integration enabled finer stratification. This advancement is crucial for proactive interventions, as it may reduce false negatives by 84% in low‐risk groups while optimizing monitoring intensity through risk‐based stratification.

## 3 Discussion

This study introduced a novel genetic proxy strategy that connects the biology of CSF biomarkers with the clinical applications of BD research. By developing cluster‐specific PRS anchored to the CSF protein‐metabolic network, we achieved three key advancements. First, we performed unsupervised clustering of 740 BD‐associated SNVs, identifying four distinct pathophysiological axes: metabolic‐imbalance, metabolic‐active, HLA+immune, and HLA‐immune clusters. Each axis exerted coordinated effects on CSF biomarkers and different clinical trajectories. Second, integrating cluster‐specific PRS and CSF biomarker PRS with machine learning improved BD risk prediction, addressing the limitations of CSF collection in clinical settings by leveraging genetically inferred biological subtypes. Third, the joint multiplicative model combining clinical scales and multimodal PRS enabled fine stratification, particularly reducing false negatives in low‐risk cohorts, and optimized monitoring intensity through risk‐tailored approaches.

Our findings suggested that CSF biomarkers, particularly those associated with immune and metabolic processes, play a significant role in BD. We identified four metabolites and 27 proteins associated with BD, which were enriched in pathways related to antigen processing, T‐cell‐mediated immunity, and immune response regulation, underscoring the potential role of immune dysfunction in BD pathology [29, 30]. Notably, the clustering analysis of BD‐related SNVs revealed coordinated genetic effects on CSF biomarkers, providing further evidence of the genetic regulation of BD‐related biological processes.

Our approach overcame the major limitations of previous biomarker studies. Cross‐sectional CSF analyses face challenges in addressing collection issues [31, 32], whereas cluster‐specific PRS captures the genetic predisposition to pathway dysregulation, explaining an additional 1.84% of the variance in BD risk beyond clinical scores. The scalability of this framework is demonstrated by its successful application to 247,834 UKB participants without CSF data, where high‐risk individuals were identified with biologically interpretable subtypes.

The immune‐ and metabolism‐related clusters identified in this study highlight two fundamental biological axes in BD pathophysiology. From a metabolic perspective, the metabolic‐imbalance cluster was associated with the downregulation of *SIRT5*, a mitochondrial sirtuin that regulates oxidative phosphorylation, consistent with autopsy evidence showing reduced synaptic mitochondrial density in the dorsolateral prefrontal cortex in BD [33, 34, 35]. This cluster displayed broad pleiotropy across 21 psychiatric phenotypes, reflecting GWAS findings linking mitochondrial dysfunction with cross‐diagnostic psychopathology [36]. The superior predictive performance of this cluster may be explained by both biological and technical factors. Biologically, it captured coordinated mitochondrial and neuroendocrine dysregulation, including reduced *SIRT5* expression and altered gonadotropin signaling, key processes influencing energy metabolism, oxidative stress [37], and the hypothalamic–pituitary axis that underlie stress reactivity and emotional regulation [38, 39]. Technically, the cluster contained a higher density of independent variants with moderate effect sizes and weaker linkage disequilibrium, enhancing the stability, generalizability, and predictive accuracy of its polygenic risk score. The selective protection of the metabolic‐active cluster against anxiety may reflect compensatory phosphatidylcholine signaling, a potential mechanism for lipid‐targeted therapies [40, 41].

In contrast, the extreme polarization of *MICB* and *SIRT5* in the HLA‐immune cluster suggested that a BD subtype characterized by immune checkpoint dysregulation and mitochondrial stress may explain the 14% increased risk of emotional instability observed in this BD subgroup [42, 43, 44]. Clinically, the HLA+immune cluster is associated with earlier onset of BD and reduced misery, suggesting an accelerated neurodevelopmental trajectory in the inflammatory subtype [45, 46]. This is consistent with neuroimaging evidence showing cortical thinning in carriers of the HLA‐C risk allele [47]. These biologically based subtypes enable precise monitoring, with individuals reclassified into the highest 2.5% risk tier by our multiplicative model showing a 17.6‐fold increase in BD incidence, surpassing the predictive capacity of the clinical model.

The limitations of this study included the fact that the primary analysis was conducted using individuals of European ancestry, and validation in diverse populations was needed. Additionally, there was a lack of comparison with directly constructed CSF biomarker risk models, although comparisons were made with risk models based on plasma proteomics. Furthermore, the number of BD cases within the UKB cohort was relatively limited compared with large‐scale psychiatric consortia, which may reduce the statistical power for certain downstream analyses.

Beyond improving statistical prediction, the cluster‐specific PRS offered clinically meaningful insights into BD heterogeneity. Each PRS represented a biologically coherent axis of risk, such as metabolic–mitochondrial dysregulation or immune checkpoint imbalance, allowing genetic risk to be interpreted in a mechanistic context. This framework enabled risk stratification based not only on genetic burden but also on underlying pathophysiological pathways, which could inform early monitoring strategies and guide targeted interventions. For instance, individuals with high metabolic‐imbalance PRS may benefit from preventive monitoring of metabolic and endocrine function, whereas those with elevated HLA‐related PRS might represent an inflammatory subtype that could respond to immunomodulatory approaches. Together, these findings illustrated how biologically informed PRS could bridge molecular genetics and clinical application in BD precision medicine.

## 4 Conclusions

This work advanced BD research in several ways. First, it delineated a genetically anchored CSF biomarker network. Second, it demonstrated the clinical utility of integrating multimodal PRS. Finally, it provided a scalable framework for BD risk stratification. These insights paved the way for precision monitoring and therapeutic strategies tailored to the heterogeneous pathophysiology of BD.

## 5 Methods and Materials

### 5.1 GWAS Summary Statistics

The BD GWAS summary data come from the largest GWAS meta‐analysis to date, including 214,196 valid samples from European populations (57,833 BD patients and 722,909 controls) [48]. The CSF proteome data consist of 3107 samples from six cohorts, covering 1786 unique proteins and 2316 index pQTL associations [9]. The CSF metabolome includes data from 2602 samples across five cohorts, encompassing 440 metabolites, with 219 association signals identified for 144 metabolites (Tables S19 and S20) [10].

### 5.2 UKB Data

UKB is a prospective cohort that recruited over 500,000 participants aged between 37 and 73 years, who underwent assessments at one of 22 assessment centers between 2006 and 2010 [49, 50]. The UKB has received approval from the National Health and Social Care Information Centre and the Northwest Research Ethics Committee of the National Health Service. We defined BD patients by the 10th revision of the International Classification of Diseases (ICD‐10, F31) [49].

Following previous studies [13], we excluded participants with sex chromosome aneuploidy, genetic relatedness, non‐European ancestry, baseline diagnosis of BD, and those with over 85% missing BD‐related questionnaire data. We retained 247,834 European ancestry samples for analysis and performed multiple imputations using Multiple Imputation by Chained Equations (MICE). The diagnostic data included anxiety disorders, BD, delusional disorders, dementia, depression, developmental disorders, disorders of sexual preference, dissociative conversion disorders, eating disorders, gender identity disorders, mental and behavioral disorders, mental retardation, obsessive‐compulsive disorder, organic mental disorders, other affective disorders, personality disorders, reaction to severe stress, schizophrenia, sexual dysfunction, and sleep disorders, as well as 13 psychological traits: mood swings (p1920), miserableness (p1930), irritability (p1940), sensitivity (p1950), fed‐up (p1960), nervous feelings (p1970), worrier/anxious feelings (p1980), tense/highly strung (p1990), worry too long after embarrassment (p2000), suffer from nerves (p2010), loneliness/isolation (p2020), guilty feelings (p2030), and risk taking (p2040) (Tables S21 and S22). Additionally, a cohort of 1593 BD patients from UKB was constructed for comorbidity studies.

### 5.3 CSF Biomarker Identification

To identify proteins and metabolites associated with BD, the FUSION was applied to construct protein (PWAS) and metabolite (MWAS) level prediction models based on significant associations [51]. These predicted protein/metabolite levels were then examined in correlation with BD, with FDR (*q* < 0.05) as multiple testing corrections. For correlated biomarkers, MR analysis was performed, with biomarkers as exposures and BD as outcome. Proteins/metabolites that were associated with BD in both approaches were included as candidate biomarkers in subsequent analyses [52]. In addition, to rule out overlap driven by linear dependency, we performed Bayesian colocalization analysis [53]. Variants located within ±250 kb of the index QTL variants were included in the analysis. A shared association was considered present when the posterior probability for hypothesis 4 (H4, indicating a shared causal signal) was ≥ 0.80 (details in Supporting Information).

### 5.4 Unsupervised SNV Clustering

Before clustering, we select the independent SNPs with *p* < 5 × 10^−6^ and *r* ^2^ < 0.6 within 1 Mb to ensure the robustness of clustering results. If the physical distance between lead SNVs was less than 500 kb, genomic risk loci were defined by merging the genomic regions [54].

Following the research by Suzuki et al. [55], we used three‐step strategy to define the functional clusters of BD using CSF GWAS summary statistics. First, we realigned the effect estimates of candidate CSF biomarkers to the BD risk allele as follows (Equation 1):

(1) $$Z_{ij}=\frac{\beta_{ij}}{\sqrt{N_{i}}s_{ij}}$$

where $\beta_{ij}$ was represented as the effect size of CSF GWAS of the jth BD SNV and the ith biomarker, $s_{ij}$ was indicated as the standard error of $\beta_{ij}$, and $N_{i}$ was the maximum sample size. Specifically, if no association summary statistics were available, the z‐score was set to “missing” [55]. Next, we used the *ClustImpute* R package to perform k‐means clustering on the SNVs and estimate the missing *z*‐scores. For a predefined number of clusters, ClustImpute randomly replaces the missing *z*‐scores from the marginal distribution of the phenotype during the first iteration and performs *k*‐means clustering. In subsequent iterations, missing *z*‐scores are updated based on the current cluster assignments, considering the correlations between phenotypes. Penalization weights are applied to the estimated values in each iteration, progressively decreasing to zero as the estimates for the missing data improve. Finally, we determined the optimal number of SNV clusters based on the majority rule of the clustering performance index from the *mclust* R package [56].

Finally, we tested the association between the jth biomarker and the BD SNVs in each cluster using linear regression (Equation 2):

(2) $$Z_{j}∼\gamma_{ik}C_{jk}$$

where $C_{jk}$ was an indicator variable, taking the value of 1 if the jth BD SNV is assigned to the kth cluster, and 0 otherwise, and $\gamma_{ik}$ was the effect size [55].

### 5.5 Cluster‐Specific PRS and Clinical Associations

Based on the cluster information, we constructed PRS with the selected SNPs. After adjusting for the top five genetic principal components (PCs), we used 247,834 UKB EUR individuals to calculate the cluster‐specific PRS ($\mathrm{PR}S_{\mathrm{Cluster}}$) (Equation 3):

(3) $$\mathrm{PR}S_{C\mathrm{luste}r_{k}}=\sum_{m=1}^{p_{k}}\beta_{m}X_{m}+\sum_{n=1}^{5}\beta_{n}PC_{n}$$

where $\beta_{m}$ is the effect size of the mth SNP of BD summary statistics, k is the cluster index (e.g., C1, C2, C3, and C4 in our study), and $p_{k}$ is the number of SNPs in cluster k. We subsequently examined the association between the $\mathrm{PR}S_{\mathrm{Cluster}}$ and psychiatric‐related phenotypes, including 20 psychiatric disorders and 13 mental health traits in the general population. Additionally, we investigated the interaction effects between three types of childhood trauma and cluster‐specific PRS on BD susceptibility [57]. Furthermore, we assessed the relationships between $\mathrm{PR}S_{\mathrm{Cluster}}$ and BD comorbidities, as well as age at onset in cohorts of BD. All association analyses were conducted using the *glm* function in R, with gender and age at first recruitment regarded as covariates. Note that we regarded age at onset of BD as a covariate for BD‐related comorbidities.

Additionally, we compared the prediction performance between $\mathrm{PR}S_{\mathrm{Cluster}}$ and overall PRS model, which was constructed based on the *auto* version of Deterministic Bayesian Sparse Linear Mixed Model (DBSLMM) (v0.3) [58, 59]. Similarly, the CSF biomarker PRS was constructed using DBSLMM. After selecting the biomarkers with positive heritability ($h^{2}$) with linkage disequilibrium score regression (LDSC), we retained 12 biomarkers, including one metabolite and 11 proteins, for PRS analysis [60]. The –*score* flag was used in PLINK2 to normalize the PRS.

### 5.6 Cox Proportional Hazards Regression Model

Individuals at baseline received psychiatric questionnaires, which took place between 2006 and 2010. Participants were considered at risk for BD from baseline and followed until the first diagnosis of BD, death, loss to follow‐up, or the last available data (April 2021), with a mean follow‐up of 12.15 years.

We constructed five Cox models, including the baseline model (gender + age), the overall PRS model (baseline model + overall BD PRS), the cluster‐specific PRS model (baseline model + cluster‐specific PRS), the machine learning hybrid model (baseline model + machine learning), and the Clinical Score model. For the machine learning hybrid model, we included 12 CSF biomarkers PRSs (1 metabolite PRS and 11 protein PRS) and C1 PRS (the best‐performing cluster from the cluster‐specific PRS). We divided the dataset into training and testing groups at a 7:3 ratio. Models were then fitted using Elastic Net (Enet), Stepwise Cox (StepCox), Ridge, and Least Absolute Shrinkage and Selection Operator (Lasso) [61, 62, 63], with *K*‐fold cross‐validation performed on the training dataset. For the Clinical Score model, we extracted four BD‐related score—ever manic/hyper for 2 days (p4642), manic/hyper symptoms (p6156), length of longest manic/irritable episode (p5663), and severity of manic/irritable episodes (p5674)—and four depression‐related scales (referred to as PHQ‐4): frequency of depressed mood in last 2 weeks (p2050), frequency of unenthusiasm/disinterest in last 2 weeks (p2060), frequency of tenseness/restlessness in last 2 weeks (p2070), and frequency of tiredness/lethargy in last 2 weeks (p2080). We regarded the clinical score as the sum of the eight indices.

All Cox models were fitted using the *coxph* function in R. We further evaluated the model's performance by calculating the corrected C‐index with 1000 bootstrapping repetitions using the *rms* package [64]. The likelihood ratio test was used to compare the model fit of nested Cox models in the testing group via the *anova* function in R. Additionally, the AIC and BIC indices for each Cox model were computed for the testing group [65].

Finally, we constructed a multiplicative model based on the testing group by multiplying the absolute clinical risk estimated from the clinical score with the relative mixed risk measured by the machine learning model to estimate the overall risk for individuals and reclassify BD risk [66]. Specifically, when the PRS value was ≤ 1, it was set to 1 to avoid negative or unstable multiplicative effects. Participants were then categorized into low‐, intermediate‐, and high‐risk groups based on the distribution of the multiplicative risk factor, using empirical cutoffs of 7 and 14, which approximately corresponded to the 20th and 80th percentiles of the distribution, respectively. We also compared the nested Cox models' goodness‐of‐fit and assessed whether the proportional hazards assumption held using the Schoenfeld residuals method.

## Author Contributions

S.H. and S.Y. conceived and designed the study. Y.F., X.G., P.H., X.J., and N.J. analyzed the data. Y.F., X.G., and P.H. interpreted the findings. Y.F., X.G., S.Y., and S.H. drafted or substantively revised the paper. All authors have read and approved the final manuscript.

## Ethics Statement

Ethical approval of UK Biobank study was granted by the National Health Service National Research Ethics Service (reference 11/NW/0382). All GWAS resources were approved by relevant ethics committees, and written informed consent was obtained from all participants.

## Conflicts of Interest

The authors declare no conflicts of interest.

## Supporting information




**Figure S1**: Alignment of 31 CSF biomarkers with 740 BD risk alleles from SNVs. The z‐scores were then adjusted based on sample size, and Pearson correlation analyses were performed between each of the 31 CSF biomarkers and BD SNVs.
**Figure S2**: Determination of the optimal number of clusters for 20 CSF biomarkers aligned with BD risk alleles using the mclust package in R.
**Figure S3**: After adjusting for the first five PCs, the BD effect size of each cluster SNV was multiplied by the genotypes of UKB individuals to calculate the Cluster‐specific PRS, which was then standardized to have a mean of zero and unit variance. The association between the four Cluster‐specific PRSs and psychiatric disorders was tested. This figure presents only the results with a P‐value <  .05.
**Figure S4**: The association between the four Cluster‐specific PRSs and psychological trait‐related phenotypes was tested. This figure presents only the results with a P‐value <  .05.
**Figure S5**: The association between the four Cluster‐specific PRSs and BD‐related comorbid psychiatric symptoms was tested. This figure presents only the results with a P‐value <  .05.
**Figure S6**: Normal distribution plot for overall PRS.
**Figure S7**: Normal distribution plot for C1 PRS.
**Figure S8**: Comparative Hazard Ratios across different predictive models.
**Figure S9**: Prediction of BD pathogenesis by individual plasma proteomics from UKB.
**Figure S10**: Schoenfeld residual test for multiplicative modeling.

**Table S1**: PWAS‐FUSION Analysis Results.
**Table S2**: MWAS‐FUSION Analysis Results.
**Table S3**: Mendelian Randomization Analysis of CSF Proteins as Exposures for BD Risk.
**Table S4**: Mendelian Randomization Analysis of CSF Metabolites as Exposures for BD Risk.
**Table S5**: Identify index pQTL associations for 61 proteins using PLINK.
**Table S6**: Identify index mQTL associations for 20 metabolites using PLINK.
**Table S7**: Colocalization analysis between index pQTL variants and BD.
**Table S8**: Colocalization analysis between index mQTL variants and BD.
**Table S9**: Enrichment Analysis of BD‐Associated CSF Proteins.
**Table S10**: 740 BD‐Associated SNVs.
**Table S11**: Associations Between CSF Biomarkers and BD‐Related SNVs Across Clusters.
**Table S12**: Associations Between Cluster‐Specific PRS and Psychiatric Disorders and Mental Health Traits.
**Table S13**: Interaction Effects Between Cluster‐Specific PRS and Childhood Trauma on BD Risk.
**Table S14**: Associations Between Cluster‐Specific PRS and Psychiatric Comorbidities in BD Patients.
**Table S15**: Cox Regression Models in the Test Set Across Different Predictive Models.
**Table S16**: Comparative Hazard Ratios Across Different Predictive Models.
**Table S17**: Cox Regression Models in the BD subtypes Across Different Predictive Models.
**Table S18**: Risk Reclassification for BD Using the Multiplicative Model.
**Table S19**: Detailed of CSF Proteins GWAS Summary.
**Table S20**: Detailed of CSF Metabolite GWAS Summary.
**Table S21**: Comprehensive Psychiatric Disorder Data in UKB After Excluding BD Cases at Baseline.
**Table S22**: Detailed Comorbidity Profile of BD Patients in UKB.

## Data Availability

The data used in this research are publicly available through the UKB, and access can be obtained via the official UKB website. GWAS summary statistics for CSF and BD are downloaded from GWAS Catalog (https://www.ebi.ac.uk/gwas/) and the Psychiatric Genomics Consortium (https://www.med.unc.edu/pgc/),
