# Supplementary material for: Cerebrospinal Fluid Genetics Enhance Risk Stratification in Bipolar Disorder
Source: MedComm (2020). 2026 Feb 26;7(3):e70629. doi: 10.1002/mco2.70629 (PMC12946660; doi:10.1002/mco2.70629)
Supplement: Supplementary file 1 — Figure S1: Alignment of 31 CSF biomarkers with 740 BD risk alleles from SNVs. The z‐scores were then adjusted based on sample size, and Pearson correlation analyses were performed between each of the 31 CSF biomarkers and BD SNVs. Figure S2: Determination of the optimal number of clusters for 20 CSF biomarkers aligned with BD risk alleles using the mclust package in R. Figure S3: After adjusting for the first five PCs, the BD effect size of each cluster SNV was multiplied by the genotypes of UKB individuals to calculate the Cluster‐specific PRS, which was then standardized to have a mean of zero and unit variance. The association between the four Cluster‐specific PRSs and psychiatric disorders was tested. This figure presents only the results with a P‐value < .05. Figure S4: The association between the four Cluster‐specific PRSs and psychological trait‐related phenotypes was tested. This figure presents only the results with a P‐value < .05. Figure S5: The association between the four Cluster‐specific PRSs and BD‐related comorbid psychiatric symptoms was tested. This figure presents only the results with a P‐value < .05. Figure S6: Normal distribution plot for overall PRS. Figure S7: Normal distribution plot for C1 PRS. Figure S8: Comparative Hazard Ratios across different predictive models. Figure S9: Prediction of BD pathogenesis by individual plasma proteomics from UKB. Figure S10: Schoenfeld residual test for multiplicative modeling. [file MCO2-7-e70629-s002.docx]

**Supplementary files for**

**Cerebrospinal Fluid Genetics Enhance Risk Stratification in Bipolar Disorder**

**Supplementary Methods**

**PWAS/MWAS**

Due to the large number of trans-pQTL associations in CSF proteins, the index variants of each pQTL association were used as reference variants. All variants within a 1MB region surrounding each index variant were included in the analysis. Weight calculation methods, including "top1," "lasso," and "enet," were employed^1^. Only regions/variants with significant pQTLs were considered for weight calculation, and a genetic association p-value threshold of 1 was set to ensure that all associated/variant pairs were included in the analysis^2^.

Metabolite phenotypes and covariates were incorporated to compute weights, excluding cohort and array covariates, as numerous covariates could interfere with MWAS and result in many tests failing^3^. Despite slight biases from cohort and array differences, the predicted metabolite levels are considered reliable due to the high importance of the associated gene regions. Each metabolite association region’s weight was calculated using all variants within a 2Mb region centered on the associated major variant. FUSION used the GCTA-GREML program to compute the heritability of each associated region^1, 4^. In the CSF MGWAS, 172 associated regions across 133 metabolites were found to be heritable based on SNPID (chr: pos: ref: alt) using FUSION (default p < 0.01). Each of these associations' weights was calculated.

Since both PWAS and MWAS were based on the GRCh38 reference genome, whereas BD GWAS was based on GRCh37, we utilized the liftOver tool to convert and standardize the data to GRCh37^5^.

**MR Analysis**

We used the SNPs proposed from the positive CSF-related biomarkers in the PWAS/TWAS-FUSION analysis as instrumental variables for Mendelian Randomization (MR). To identify SNPs significantly associated with the exposure, we set a genome-wide significance threshold of P < 5 × 10⁻⁸. If no SNPs met this threshold, we relaxed it to P < 5 × 10⁻⁶. To address potential bias due to linkage disequilibrium (LD), we set the LD criteria for SNPs significantly associated with the exposure to r² < 0.01 and a distance of > 5000 KB. We then performed two-sample Mendelian Randomization (TSMR) using the "TwoSampleMR" package in R. For causal analysis between the exposure and outcome, we used the Inverse Variance Weighted (IVW) method as the primary analysis^6^. Additionally, we employed Cochran's Q test to assess heterogeneity between the instrumental variables (IVs) and used the MR Egger intercept test to check for horizontal pleiotropy across the IVs.

**Colocalization analysis**

To exclude potential overlaps driven by linear dependency, we performed Bayesian colocalization analysis. First, QTL-associated SNVs were identified using PLINK 1.9^7^, applying a genome-wide significance threshold of P < 5 × 10⁻⁸ and selecting independent variants within ±500 kb regions with r² < 0.2.

Subsequently, Bayesian colocalization was conducted using the coloc.abf function^8^. In this framework, H3 indicates that both traits are associated but have distinct causal variants, whereas H4 represents a shared causal variant underlying both associations. Default prior probabilities were used (p_1_ = p_2_ = 1 × 10⁻⁴, p_12_ = 1 × 10⁻⁵), where p₁ and p₂ denote the prior probabilities that a SNP is associated with trait 1 and trait 2, respectively, and p_12_ represents the prior probability that a SNP is jointly associated with both traits.

**References**

1. Gusev A, Ko A, Shi H, et al. Integrative approaches for large-scale transcriptome-wide association studies. *Nat Genet*. 2016;48(3):245-52.

2. Western D, Timsina J, Wang L, et al. Proteogenomic analysis of human cerebrospinal fluid identifies neurologically relevant regulation and implicates causal proteins for Alzheimer's disease. *Nat Genet*. 2024;56(12):2672-2684.

3. Wang C, Yang C, Western D, et al. Genetic architecture of cerebrospinal fluid and brain metabolite levels and the genetic colocalization of metabolites with human traits. *Nat Genet*. 2024;56(12):2685-2695.

4. Yang J, Lee SH, Goddard ME, Visscher PM. GCTA: a tool for genome-wide complex trait analysis. *Am J Hum Genet*. 2011;88(1):76-82.

5. Genovese G, Rockweiler NB, Gorman BR, et al. BCFtools/liftover: an accurate and comprehensive tool to convert genetic variants across genome assemblies. *Bioinformatics*. 2024;40(2)

6. Lv X, Xu B, Tang X, et al. The relationship between major depression and migraine: A bidirectional two-sample Mendelian randomization study. *Front Neurol*. 2023;14:1143060.

7. Chang CC, Chow CC, Tellier LC, Vattikuti S, Purcell SM, Lee JJ. Second-generation PLINK: rising to the challenge of larger and richer datasets. *Gigascience*. 2015;4:7.

8. Giambartolomei C, Vukcevic D, Schadt EE, et al. Bayesian test for colocalisation between pairs of genetic association studies using summary statistics. *PLoS Genet*. 2014;10(5):e1004383.

**Supplementary Figures**

**
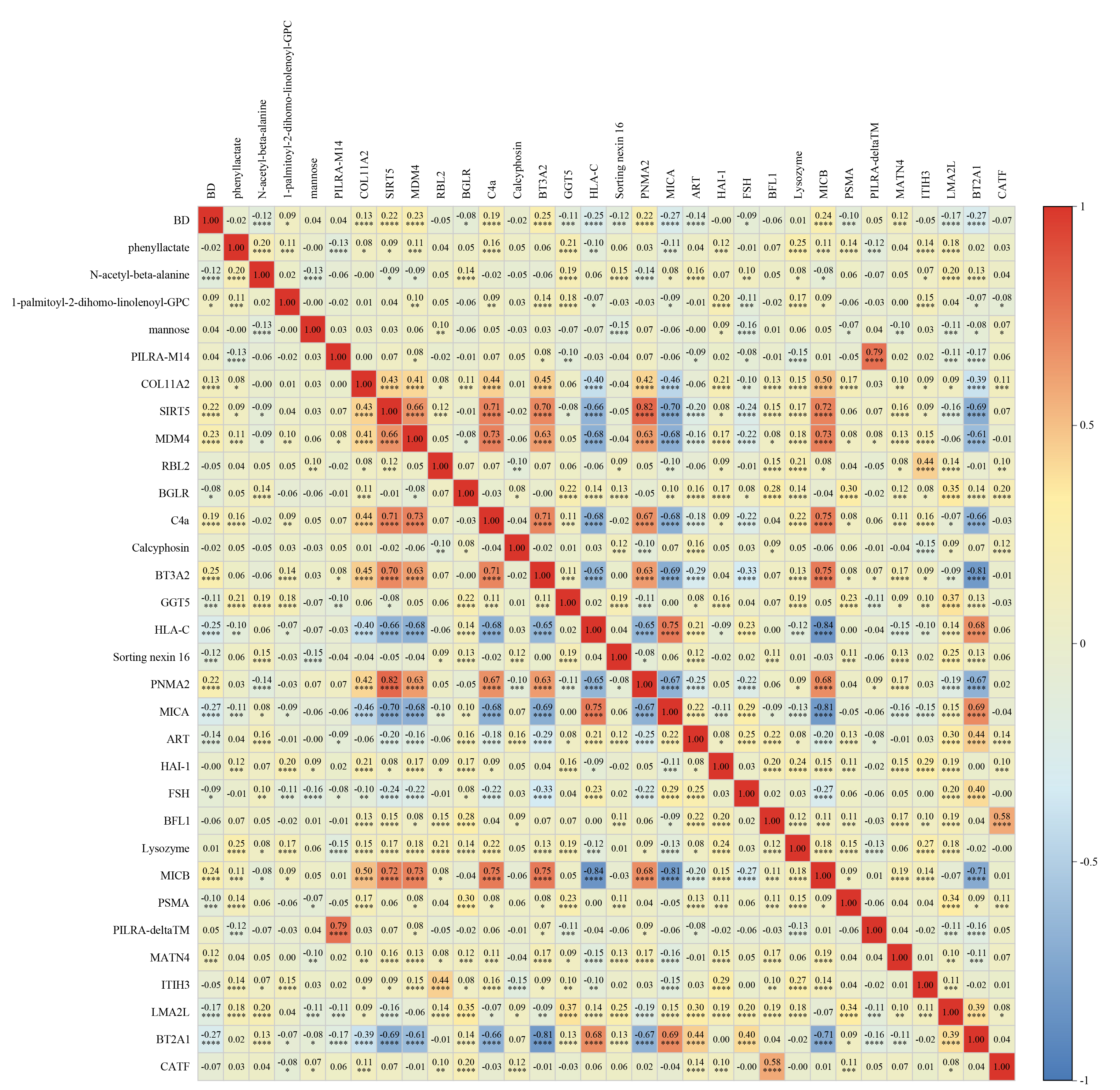
**

**Figure S1.** Alignment of 31 CSF biomarkers with 740 BD risk alleles from SNVs. The z-scores were then adjusted based on sample size, and Pearson correlation analyses were performed between each of the 31 CSF biomarkers and BD SNVs.

**
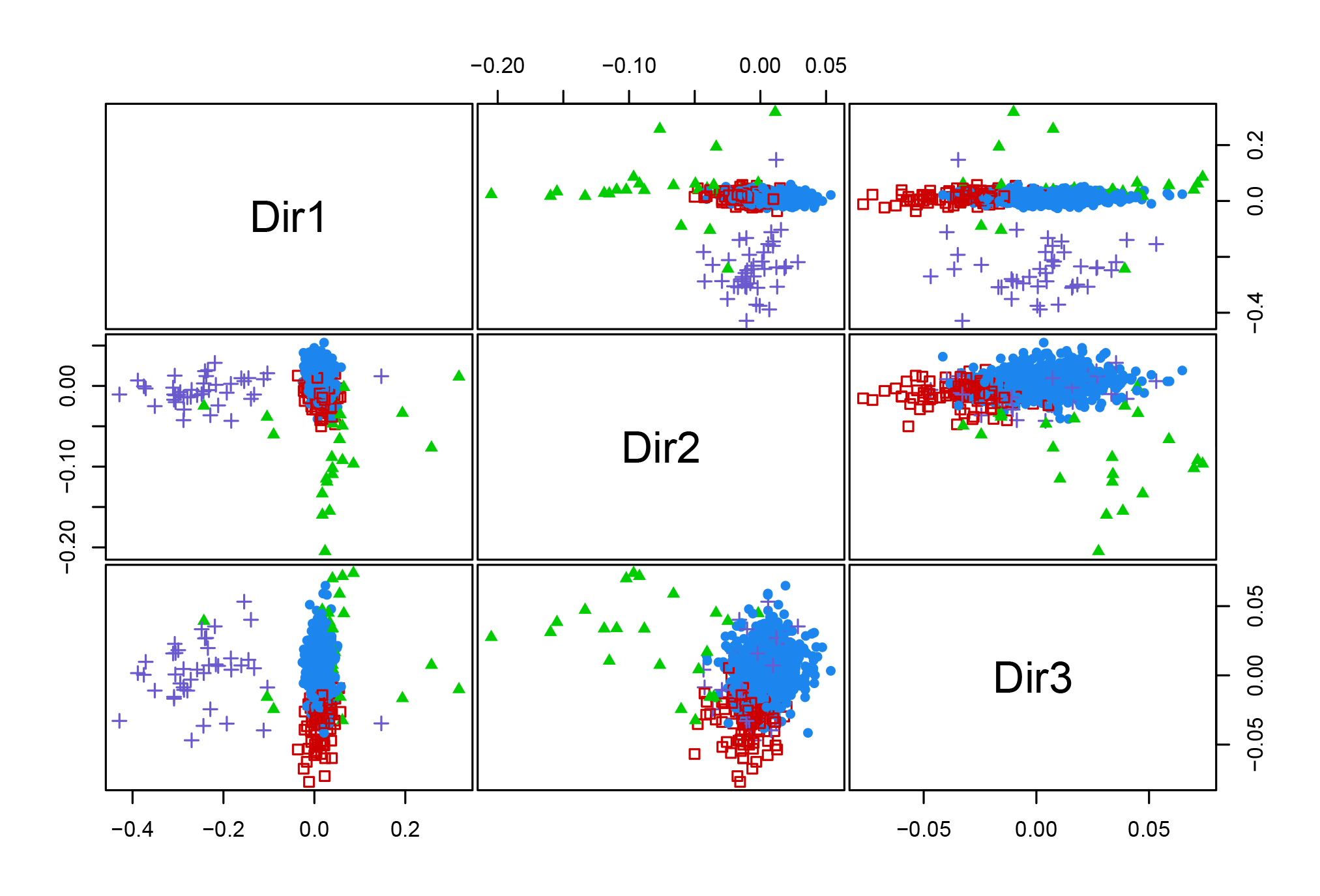
**

**Figure S2.** Determination of the optimal number of clusters for 20 CSF biomarkers aligned with BD risk alleles using the mclust package in R.

**
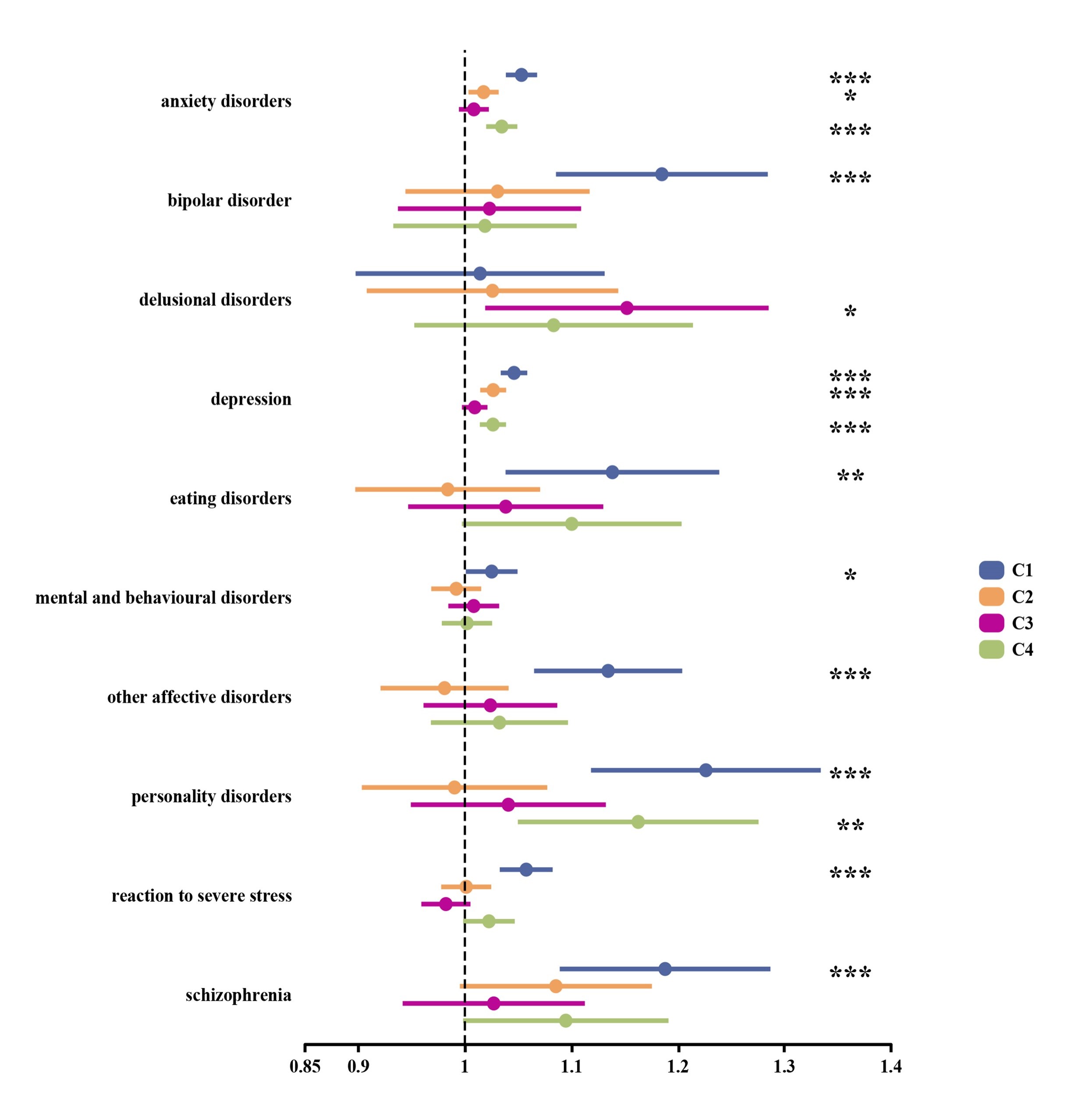
**

**Figure S3.** After adjusting for the first five PCs, the BD effect size of each cluster SNV was multiplied by the genotypes of UKB individuals to calculate the Cluster-specific PRS, which was then standardized to have a mean of zero and unit variance. The association between the four Cluster-specific PRSs and psychiatric disorders was tested. This figure presents only the results with a P-value < .05.

**
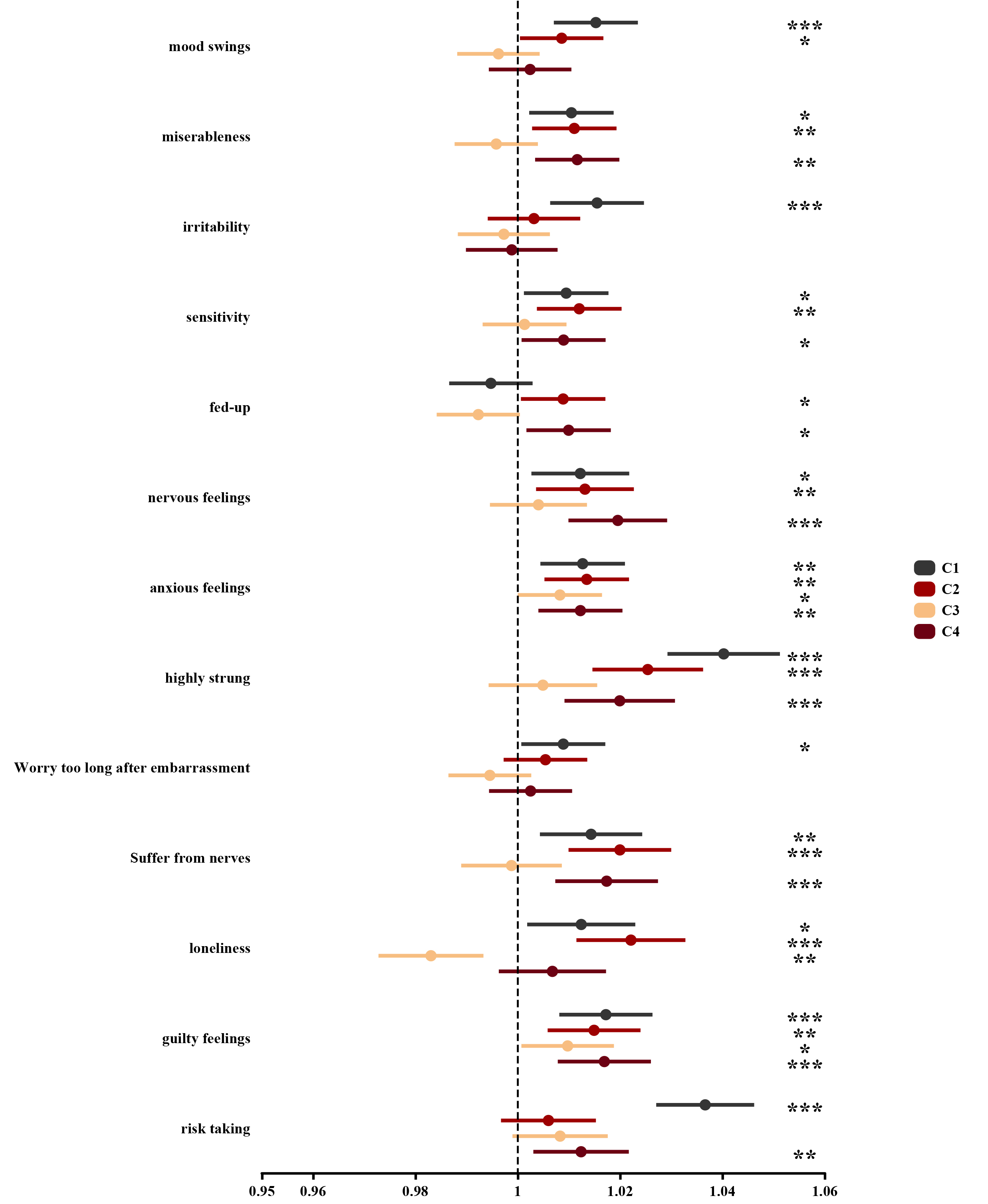
**

**Figure S4.** The association between the four Cluster-specific PRSs and psychological trait-related phenotypes was tested. This figure presents only the results with a P-value < .05.

**
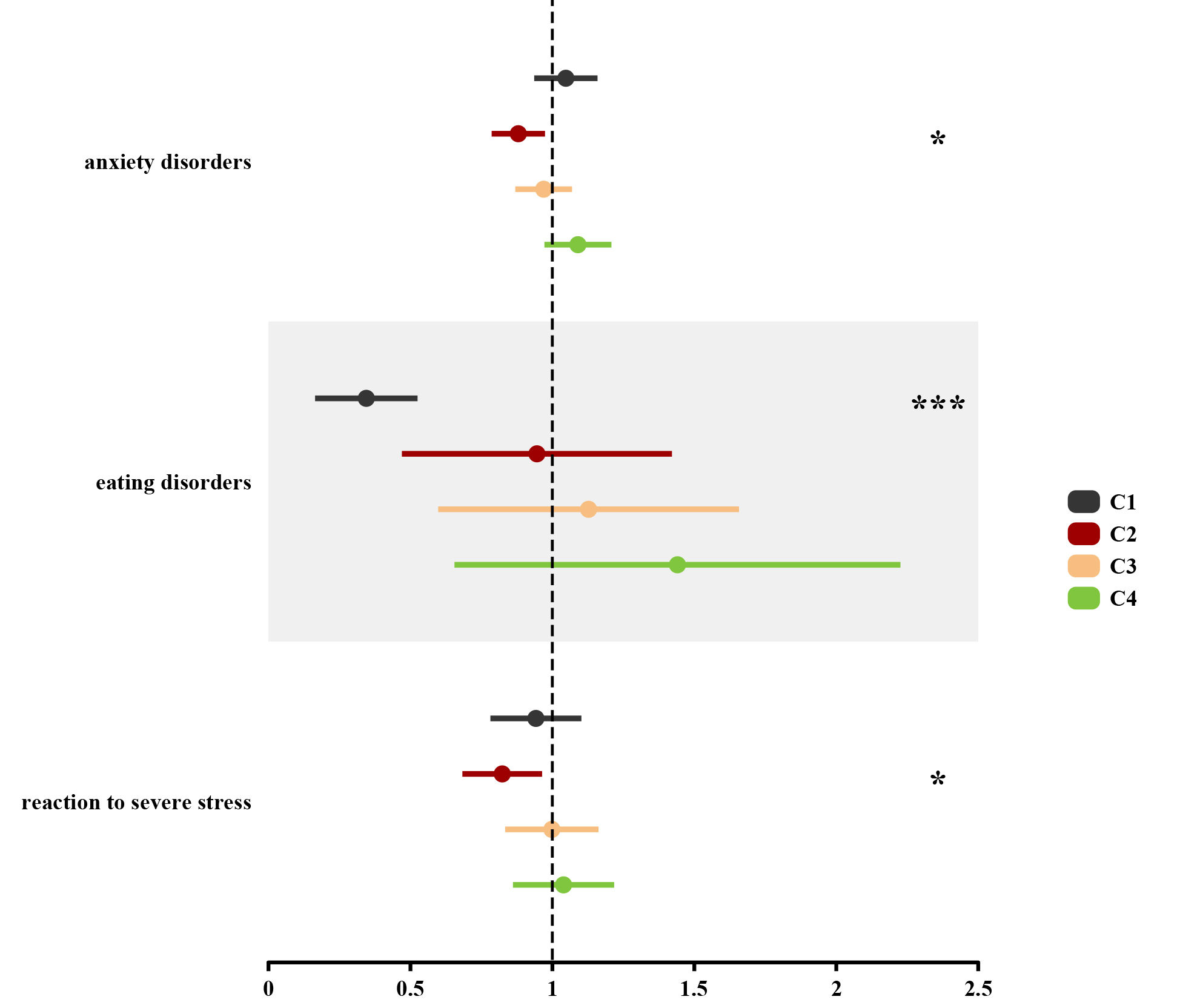
**

**Figure S5.** The association between the four Cluster-specific PRSs and BD-related comorbid psychiatric symptoms was tested. This figure presents only the results with a P-value < .05.

**
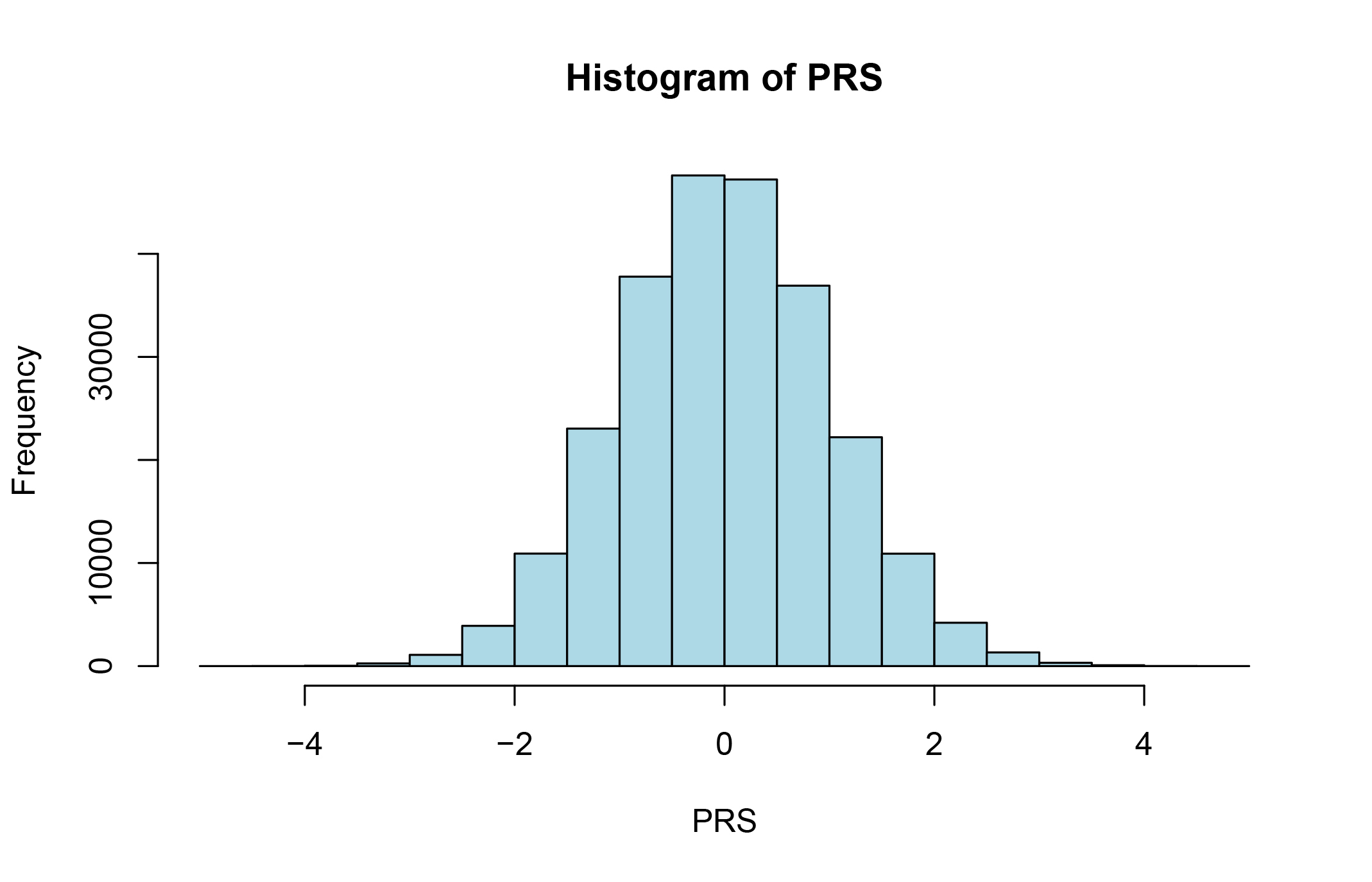
**

**Figure S6.** Normal distribution plot for overall PRS.

**
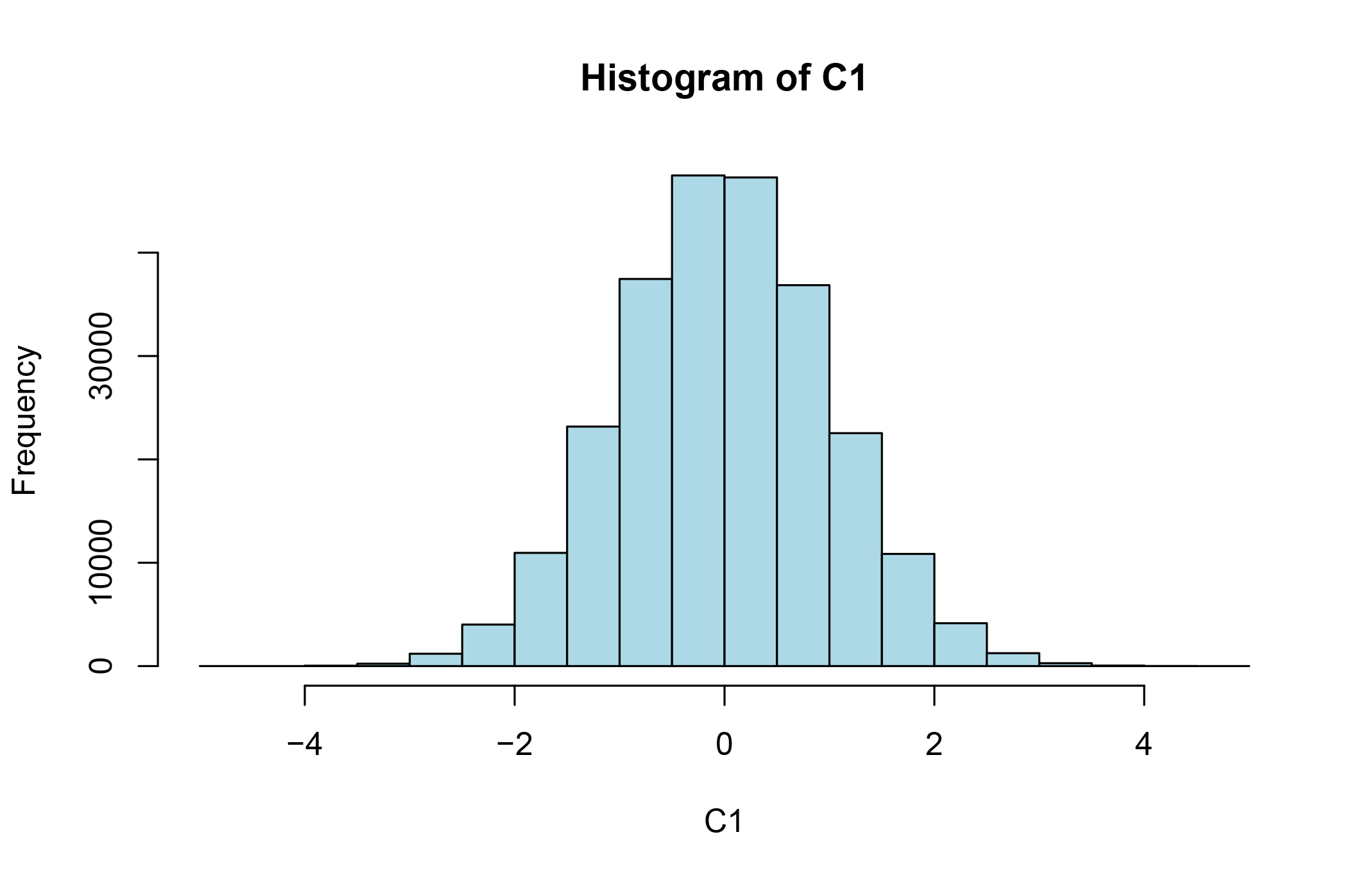
**

**Figure S7.** Normal distribution plot for C1 PRS.

**
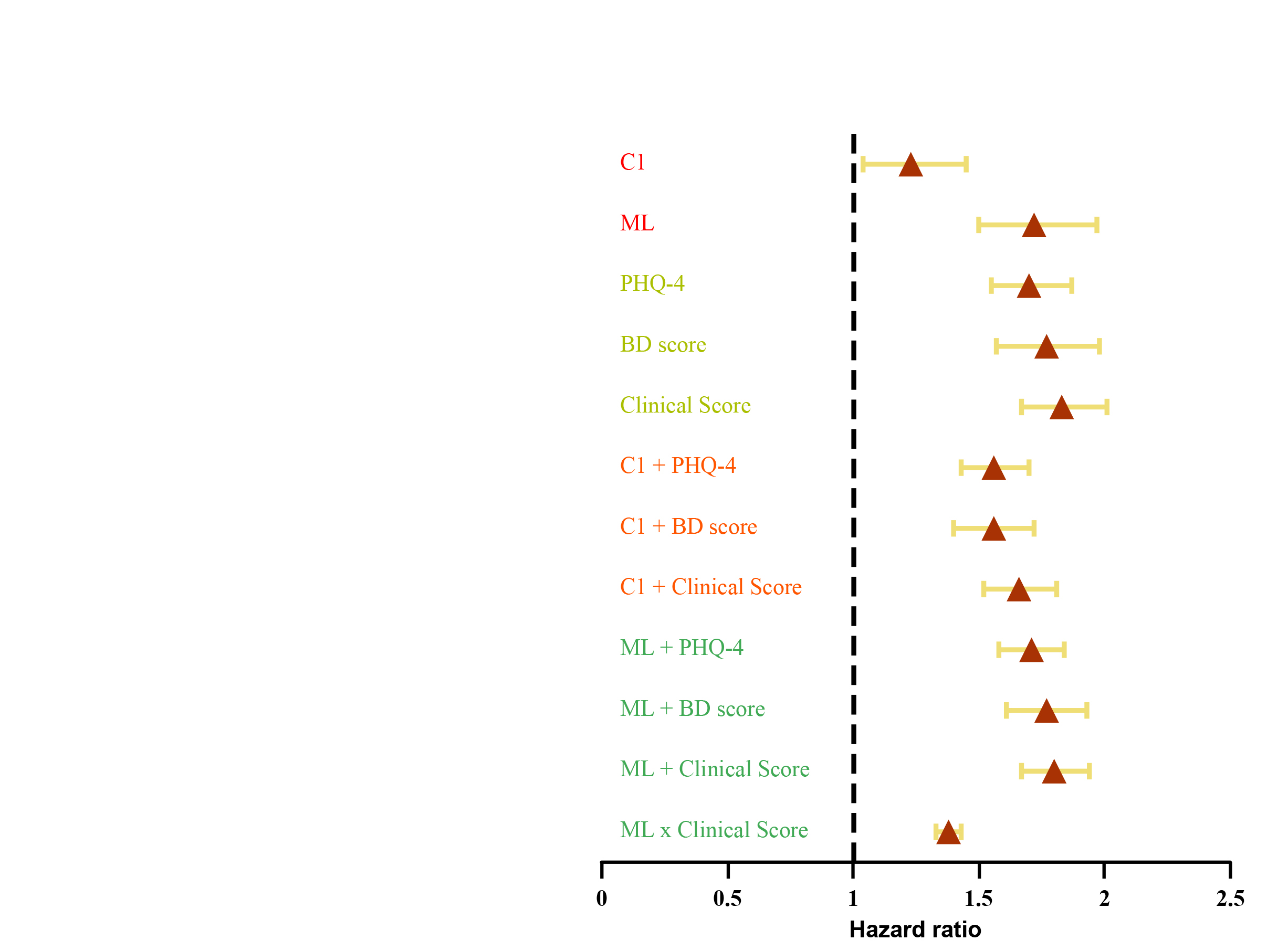
**

**Figure S8.** Comparative Hazard Ratios across different predictive models.

**
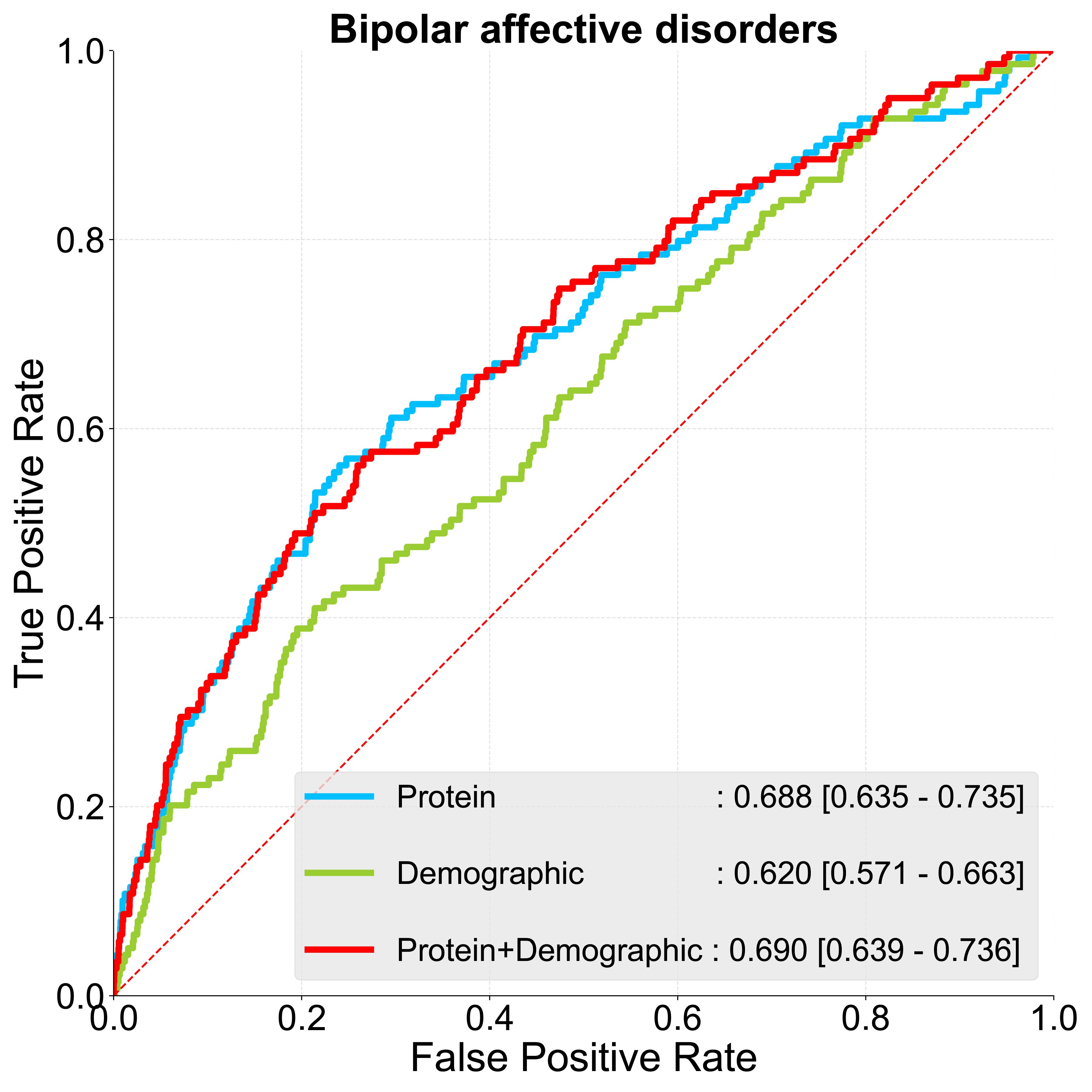
Figure S9.** Prediction of BD pathogenesis by individual plasma proteomics from UKB.

**
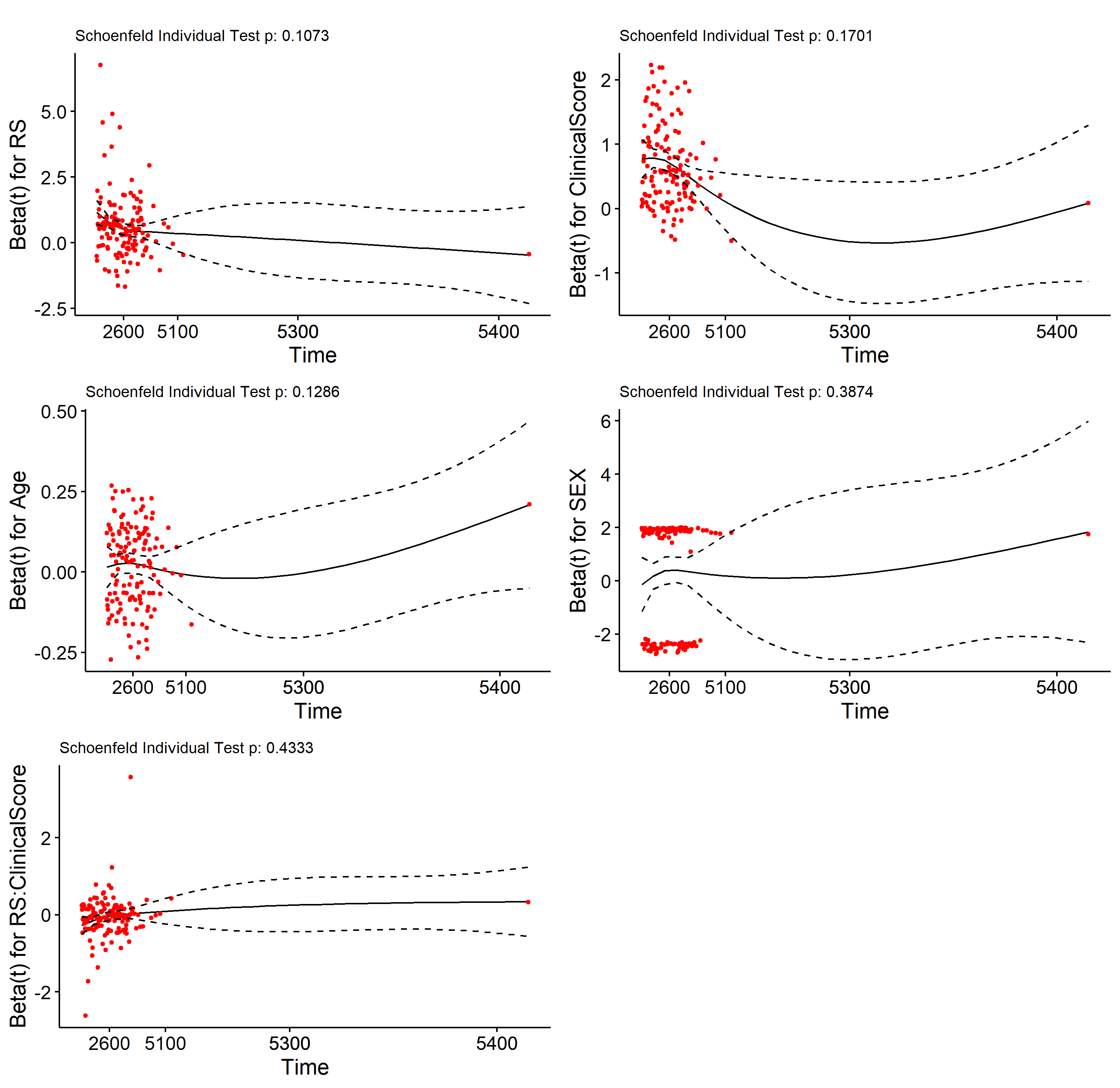
**

**Figure S10.** Schoenfeld residual test for multiplicative modeling.
